# May the Best Molecule Win: Competition ESI Mass Spectrometry

**DOI:** 10.3390/ijms161024506

**Published:** 2015-10-15

**Authors:** Sarah Laughlin, W. David Wilson

**Affiliations:** Department of Chemistry, Georgia State University, Atlanta, GA 30303, USA; E-Mail: slaughlin1@student.gsu.edu

**Keywords:** competition, mass spectrometry, electrospray, minor groove binder, DNA, transcription factor, specificity

## Abstract

Electrospray ionization mass spectrometry has become invaluable in the characterization of macromolecular biological systems such as nucleic acids and proteins. Recent advances in the field of mass spectrometry and the soft conditions characteristic of electrospray ionization allow for the investigation of non-covalent interactions among large biomolecules and ligands. Modulation of genetic processes through the use of small molecule inhibitors with the DNA minor groove is gaining attention as a potential therapeutic approach. In this review, we discuss the development of a competition method using electrospray ionization mass spectrometry to probe the interactions of multiple DNA sequences with libraries of minor groove binding molecules. Such an approach acts as a high-throughput screening method to determine important information including the stoichiometry, binding mode, cooperativity, and relative binding affinity. In addition to small molecule-DNA complexes, we highlight other applications in which competition mass spectrometry has been used. A competitive approach to simultaneously investigate complex interactions promises to be a powerful tool in the discovery of small molecule inhibitors with high specificity and for specific, important DNA sequences.

## 1. Introduction

The continued development of electrospray ionization (ESI) methods and the extensive improvements in commercial mass spectrometry (MS) instruments over the last two decades have brought ESI-MS experiments into the forefront of analysis of biomacromolecules and their complexes. The majority of ESI-MS (electrospray ionization mass spectrometry) reports on biological systems to date have involved proteins but nucleic acids are attracting increasing attention. Early studies on double helical DNA involved establishing conditions for preserving the duplex in the gas phase and evaluating different volatile solution buffers/salts for optimum ESI-MS conditions [1]. There are numerous reports on duplex stability [1,2,3], dissociation to single strands [4,5,6], effects of ESI conditions [7,8], solution composition [9,10,11,12] and nucleic acid sequence [13,14]. These key studies and more have provided a strong foundation for ESI experiments on nucleic acid-small molecule complexes.

### 1.1. Small Molecule Studies by ESI-MS

The early studies on ESI-MS requirements for stable duplex (for example Smith and co-workers [1,15]) quickly evolved into important studies on DNA complexes with metal ions [5,16], organic compounds [17,18,19,20,21,22] and proteins [23]. The studies of organic systems complement extensive solution biophysical studies that have two important goals: (i) develop a better understanding of the fundamental features of nucleic acid interactions and (ii) design nucleic acid targeting agents for biotechnology and therapeutics. Compounds that bind in the DNA minor groove have a variety of structural features that affect their affinity, stoichiometry and sequence specificity. All of these features, as well as cooperativity for compounds with a stoichiometry greater than one, can be investigated by ESI-MS methods. The earliest reports of DNA complex by ESI-MS were with the polyamide minor groove binder, distamycin A [15,24]. The complex was intact in the gas phase and gave a 1:1 binding stoichiometry for a 12 base pair duplex that had an AAATTT base sequence binding site at low ratios of distamycin to DNA complex. As the ratio of distamycin to duplex was increased, a 2:1 bound species was observed and these results are in agreement with solution experimental findings [25,26,27]. The excellent agreement between species present and their ratio dependencies was a significant example that showed nucleic acid complex stoichiometry, cooperativity and relative affinities could be determined by ESI-MS.

The successful, initial studies of small molecule-DNA complexes moved into ESI-MS experiments with a well-known variety of agents, including organic intercalators, such as ethidium bromide [28,29,30], metallo-intercalators [31], bis-intercalators [32,33] and a wide variety of minor groove binders of quite different structure [28,34]. All of the results with minor groove binders and intercalators, for which solution results were available, were in excellent agreement with the solution studies. It is now quite clear that ESI-MS experiments, when properly conducted, will provide complementary and very useful results for analysis of DNA-small molecule complexes. The method is quite versatile and experiments can be rapidly conducted so that it is an attractive addition to other powerful, biophysical approaches for DNA complex analysis. The ability to quickly analyze non-covalent complexes, with good sensitivity and low sample consumption, as well as the variety of information provided, makes ESI-MS a very valuable tool.

### 1.2. Application to Other Systems

ESI-MS is often used for characterizing small, organic molecules but has become a powerful tool for large biomolecular systems. For small molecule-DNA investigations, excellent consistency using ESI-MS and other biophysical methods has been found. Competition ESI-MS is particularly appealing since multiple interactions between ligand and DNA can be simultaneously analyzed. Analyzing interactions between a single DNA and a single ligand is not efficient for screening a library of compounds. With a competition method, a large number of interactions can be studied in much less time than with the conventional approach of one DNA and one ligand. Valuable information is gathered quickly with regards to preferential binding of a ligand to DNA. Although several of the studies described later will focus primarily on the binding of small molecule ligands to DNA, this technique is not limited to these specific interactions and can be applicable to other biomolecules of interest, including proteins [2,22,35,36,37,38], carbohydrates [39,40], and other types of nucleic acids such as RNA [41,42,43,44,45,46,47,48,49,50,51] and peptide nucleic acids [52,53,54].

## 2. Protein Complexes with Nucleic Acids by ESI-MS

### 2.1. Transcription Factor Proteins

The ETS family of transcription factors (TF) comprises a major class of transcriptional regulators across many species, including humans [55]. Humans also express various oncogenic mutations of the ETS TFs that are associated, for example, with bone, breast, and prostate tumors [56,57]. All ETS TFs have similar DNA binding domains that are highly conserved in structure with a 5′-GGAA/T-3′ consensus central binding site [58]. Genomic analyses have identified the ETS member PU.1 as a pioneering transcription factor [59] that can overcome chromatin packaging to bind chromosomal DNA. The PU.1 TF is a unique protein, encoded by the SPI1 gene, which has high affinity binding for purine-rich DNA motifs, or PU-box. Because of the special properties of PU.1, it is important to understand how DNA recognition by PU.1 is differentiated from other ETS proteins. There is, thus, an essential need for a broad range of methods and studies, including ESI-MS, to address the physical mechanisms of sequence recognition by the PU.1 TF.

The importance of PU.1 and the many broad-based biophysical and genetic studies on PU.1 [58,60,61,62] made it an attractive choice for early ESI-MS studies to test detection of protein-DNA complexes. The goal was to find an additional, powerful method to help understand PU.1-DNA interactions. Smith and co-workers [63] used both a gel electrophoretic mobility shift assay (EMSA) and ESI-MS methods to characterize the PU.1 association with both a wild type 17 base pair DNA as well as a very similar sequence with mutations in the critical GGAA binding site of PU.1. The EMSA analysis showed strong binding of PU.1 to the wild type DNA but no detectable binding to the mutant sequence. They developed ESI-MS conditions to detect duplexes of both DNA sequences and collected very useful spectra of a PU.1 1:1 complex with the wild type DNA. The stability and amount of the protein-DNA complex were very impressive, especially for such an early study. They also did a competition binding study of PU.1 with both the wild type and mutant DNAs. In very encouraging results, which are in excellent agreement with the EMSA experiments, no significant mutant complex could be detected, even in the presence of a large excess of the mutant DNA. This result clearly shows the power of ESI-MS to characterize protein-DNA complexes and resolve differences in binding affinity that are in good agreement with other biophysical studies.

### 2.2. Protein Inhibition

Targeting TF-DNA complexes, either for inhibition or enhancement, is very attractive for the treatment of a number of different diseases. This could be done by targeting the TF or the DNA binding domain. Unfortunately, it has proved very difficult to target TFs and they are frequently referred to as “undruggable” [64,65]. An alternative is to target the DNA binding domain of the TF with designed small molecules and this approach is gaining increasing attention. As described above, ESI-MS has developed into a very attractive method to evaluate both small molecule binding to specific DNA sequences as well as for their effects on TF-DNA complexes. Ralph, Beck and co-workers conducted a very impressive illustrative example of this approach with the PU.1 protein-DNA complex [66]. They combined the PU.1 DNA binding site with the critical GGAA sequence along with the PU.1 DNA binding domain. In the positive ion ESI-MS mode, conditions were developed with the PU.1-DNA complex as the major species and small peaks for the free DNA and PU.1. They noted that similar results were also obtained in the negative ion mode. They next added Ru and Pt based DNA intercalators to the system and obtained very impressive inhibition results for the PU.1-DNA complex. In a titration of an intercalator into the TF-DNA complex, the PU.1-DNA complex peak decreased and peaks for the free protein and DNA increased. It is also impressive that they were also able to see peaks for complexes of the intercalators to protein-free DNA. They saw no evidence that the intercalator could bind to the protein. The wealth of important information obtained in this fairly simple, single experiment clearly illustrates the power of ESI-MS in both the characterization of TF-DNA complexes and in the discovery and development of TF inhibitors. Although ESI-MS studies of protein-DNA complexes are relatively rare, a number of studies have appeared in recent years and it is likely that many more are in progress.

## 3. Methods and Notes

Electrospray ionization mass spectrometry is an ideal method for analysis of biomacromolecules because of the versatility and ease it provides in characterizing interactions. Information such as stoichiometry, cooperativity, and relative binding affinities are determined quickly within a single sample solution at low concentrations. The gentle ionization technique essentially allows non-covalent interactions to remain intact. DNA, protein, or any system of interest is injected into the electrospray ionization source and directed through a capillary tube at high voltage potential. Depending on the applied potential, the solution is then aerosolized into a fine spray of charged droplets. It is believed that the solvent evaporates from the presence of a warm, dry stream of an inert gas. While the size of the droplet decreases, the charge density on the droplet surface increases. Eventually, the Rayleigh limit, or maximum charge density a droplet can have, is reached and the droplet disperses into even smaller droplets. This process repeats many times until the solution is evaporated and the formed ions are then carried towards the mass analyzer [67,68].

The ESI-MS injection process to move samples from the solvation to the gas phase produces charged species with multiple charge states during the injection ionization process. Depending on the type of species being analyzed, the ion mode analysis may vary from system to system and may depend on the net charge of the largest system present. For instance, when analyzing DNA and small molecule interactions, the overall negative net charge of the DNA is greater than the one or two positive charges on the small molecules. Therefore, negative ion mode is used for DNA-small molecule analyses. On the other hand, if one were to analyze a TF-DNA interaction, depending on the overall charge of the protein, negative or positive ion mode can be used. Either ion mode can offer insight into specific interactions that the other mode cannot detect [34] and should therefore be considered. It is best to perform experiments with both ion modes and different experimental parameters, such as voltages, temperature and RF lens tuning, when beginning studies on new systems.

For every system multiply charged species are found. For small molecule complexes with DNA in the under 20 base pair range, the most common charge states of the DNA and their corresponding small molecule complexes range from −3 to −6 in negative ion mode. Lower net charges imply the phosphate backbone of the DNA becomes partially neutralized during the electrospray process. Ammonium acetate is the preferred buffer for ESI-MS analyses in part because of its volatility and its ability to help neutralize the backbone. This is believed to occur through a proton transfer from an ammonium ion to the DNA backbone and the creation of volatile ammonia. [1,9,24,69] The amount of backbone neutralization that occurs varies based on many factors, including but not limited to, DNA size, ammonium acetate concentration, and the instrument conditions [28,29,70,71]. It is worth noting that although the presence of a positive ligand helps in neutralizing the net charge of the phosphate backbone, it typically does not affect the net charge of the DNA-small molecule complex in the gas phase.

## 4. Designing Systems for Competition ESI-MS

In design studies of new, sequence specific compounds to target promoter DNA and inhibit TF binding development of a high-throughput screening technique to simultaneously evaluate multiple features of small molecule-DNA complex interactions would be a significant advance. In this review we will describe progress in ESI-MS for detection of complexes formed between DNA and sequence specific minor groove binding compounds. The interactions are screened with a variety of designed, synthetic minor groove binders and specific DNA sequences. The complexes are characterized by the differences in DNA sequence and/or ligand structure which influence the relative binding affinity, stoichiometry, and binding mode(s). This is a true competitive assay since the strongest binding compounds will interact with available binding sites first. Secondary or non-specific interactions may then occur between sequences and the compound through weaker binding interactions.

### 4.1. Hairpin DNA Sequences

**Figure 1:**
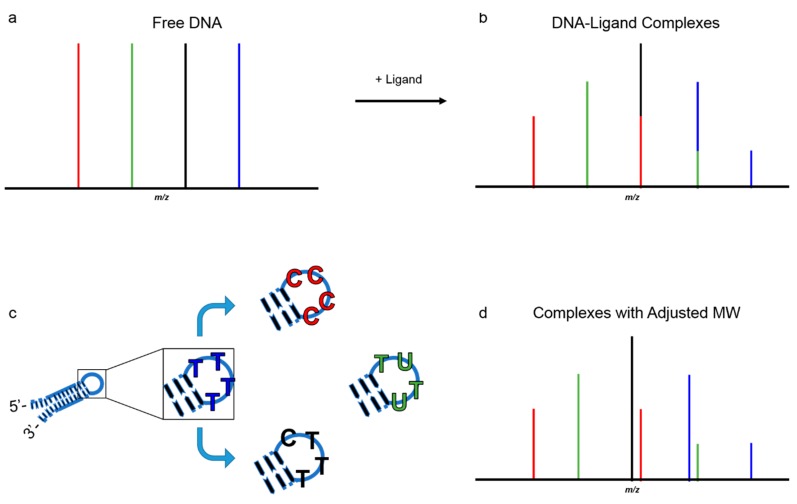
Cartoon to illustrate the adjustment of hairpin DNAs to achieve different molecular weights for ESI-MS studies. Target base pair sites in the stem of the DNA are preserved. (**a**) DNA with unadjusted molecular weights; (**b**) complex and unbound DNA peaks are not distinguishable upon addition of ligand due to overlapping peaks for free DNA and DNA-ligand complexes (e.g., black and red); (**c**) modifications in the hairpin loop by incorporation of various bases allows the DNA stem to be preserved while creating distinguishable molecular weights; (**d**) complexes and free DNA become easily identifiable. T = thymidine, C = cytidine, U = deoxyuridine.

ESI-MS is a mass-specific technique; therefore, there are many considerations in choosing the ligand and target DNA sequences. Sequences can be designed so that the base pairs adjacent to, or flanking, the target site in the stem are conserved. To do this, a single-stranded DNA sequence is designed which will fold back on itself through a self-complementary, intramolecular interaction which results in the formation of a loop on one end while the 5′ and 3′ terminal bases are hydrogen bonded at the other end (Figure 1). These secondary structures are commonly referred to as hairpin or stem loops, as they tend to resemble the loops found in devices used to secure long hair. Hairpin DNAs are the preferred structures for many studies as a dsDNA mimetic due to their simplicity (one strand *vs.* two for complementary strands) and structural stability at high temperatures and in gas phase conditions [72]. Since distinguishable molecular weights are necessary for all species and (potential) complexes involved, the hairpin loops can also be modified to incorporate different combinations of bases to adjust the molecular weights as desired since bases in the loops are not involved in binding interactions.

The molecular weights of cytidine (C), deoxyuridine (U), and thymine (T) are all different which is how the total molecular weight for a hairpin DNA would be adjusted, assuming no other modifications were made to the constant stem portion of the DNA. Figure 1 presents an example of how hairpin loop modifications offer an advantage to competitive DNA analyses. In Figure 1a, four different sequences are combined into a single sample and analyzed by ESI-MS. Let us assume, for all intents and purposes, the molecular weights for the DNA sequences are arbitrary and each sequence differs only by the addition of a base pair. The peaks for the individual DNAs are illustrated as red, green, black, and blue with the red peak corresponding to the lowest molecular weight sequence (x base pairs), green has the next lowest molecular weight (x + 1 base pairs), the black DNA has x + 2 base pairs, and the blue peak corresponds to a sequence with the highest molecular weight (x + 3 base pairs), shown in Figure 1a. Ligand is next added to the sample and DNA-ligand complexes are formed (Figure 1b). Unfortunately, the molecular weight of the ligand is similar to that of a base pair, and the molecular weights for the DNA-ligand complexes were not predetermined. Figure 1b illustrates a complex formed by ligand and red-DNA, but the molecular weight for the complex is the same as the molecular weight for unbound black-DNA (no complex). The peaks, therefore, overlap and neither species can be distinguished.

Now, let us assume the hairpin loops have been adjusted so that regardless if a DNA-ligand complex is formed, peaks will not overlap. The loops have been modified so instead of thymidine (5′-TTTT-3′) it can become all cytidines (5′-CCCC-3′), contain both cytidine and thymidine (5′-CTTT-3′) or include deoxyuridine (5′-TUTU-3′). This type of modification allows the target binding site(s) in the DNA stem to be maintained while circumnavigating the issue of overlapping peaks and molecular weights. Now, complexes formed between the ligand and red-DNA no longer overlap that of unbound black-DNA and become easily distinguishable (Figure 1d). As a side note, modifications are not limited to thymidine, cytidine or deoxyuridine and can include halogenated bases, 3′ terminal phosphate additions, *etc.* The important feature is that each base has a unique molecular weight and when combined together act as a signature for a specific DNA sequence. A very similar approach has been used by numerous groups to investigate DNA quadruplexes formed by folding of a single strand of DNA into a four-stranded structure with three connecting loops that can have a wide variety of conformations. There have also been numerous uses of ESI-MS to investigate compounds that interact with quadruplexes, for example by Beck, Brodbelt, and Gabelica [69,73,74].

### 4.2. Response & Sensitivity of DNA and DNA-Small Molecule Complexes

In ESI-MS, as well as other experiments, every analyte (small molecule, DNA, protein, *etc.*) has its own, unique physiochemical properties which contribute to the overall sensitivity or response detected. This feature is important to consider in quantitative analyses. Gabelica and co-workers formulated an additional fitting parameter, commonly referred to as the response factor, which takes into account variations in the sensitivity for each individual species detected [75]. Preservation of the flanking sequences and mutation specifically at the target binding site keeps the base pair composition and gives similar sensitivities to all of the DNA sequences and their complexes. Due to this, a direct comparison of the relative binding affinities and binding modes for the DNA-ligand complexes and free DNA is possible so factors, such as response factors, are not a significant limitation for qualitative and semi-quantitative analyses. This independence of response factors require careful design of the systems being compared, as described above.

Competitive interactions between several DNA sequences and ligands help to establish an understanding of the selectivity for potential drug candidates. One approach to examine selectivity uses a standard reference system. For instance, a non-covalent complex with a known binding constant can be included in the sample. The peak intensity of the known system can then be directly compared to the unknown species to estimate the relative binding affinities [76]. Wortmann and co-workers determined the binding affinities of ligand-protein kinase interactions using a reference system having a known binding constant [35]. This example of competition binding by ESI-MS using a known system to determine the relative binding affinities of unknown ligands can extend even into fento- and nanomolar ranges. Kempen and co-workers also employed a similar method in which the concentrations of a reference protein and ligand were held constant, while the concentration of unknown ligand was varied so that the binding constants of the unknown ligands were determined [77]. These studies are prime examples for using ESI-MS and the addition of an internal standard or reference system to determine apparent binding affinities of small molecule and DNA or protein interactions.

### 4.3. Limitations in Quantitation of DNA-Small Molecule Complexes

There are many examples in the literature, for example, by Gabelica and co-workers [75,78] which show results obtained by ESI can be accurately quantified. Unfortunately, due to the intrinsic nature of dicationic diamidines (and likely with other ligands) analyses can be limited to qualitative and semi-quantitative information such as relative binding affinities. Due to their intrinsic properties, some ligands will be lost inside the tubing during the injection process, therefore, reducing the total ligand concentration. Typically, to remove residual bound or “sticky” compounds, an excess of DNA is used to bind the ligand, followed by an extremely thorough cleaning of the instrument. Fortunately, the free ligand concentration for binding DNA remains the same within a given sample and encourages competitive binding so that preferential binding sites are easily determined. Assuming the *K*_D_ (dissociation constant) is known for a single DNA-small molecule complex, unbound and bound DNA complexes can be directly compared for relative binding affinities, and if the stoichiometry is greater than 1:1, cooperative or non-specific interactions can also be determined. Theoretically, any number of DNA sequences can be screened for binding a particular ligand which greatly reduces the volume of reagent(s) used and the amount of time spent cleaning the instrument between runs.

## 5. Competition ESI-MS

Competition ESI-MS is a frequently useful addition to existing ESI-MS techniques because it uses multiple substrates (nucleic acids, proteins, *etc.*) to screen for binding interactions with other species (small molecules, proteins, *etc.*). For example, multiple nucleic acid sequences can be screened to evaluate the relative binding affinities, stoichiometries, cooperativity and selectivity with small ligands, proteins or both at the same time. Beck and co-workers used a similar approach to investigate the competitive binding of a ruthenium compound against daunomycin and distamycin to determine the binding mode of the ruthenium to DNA [79]. Many differences in binding have been determined for a compound and its analogs using DNA sequences with systematic variations [80,81]. This method has been useful in comparing the effects of structural modification to a parent compound and its influence on DNA recognition and has become invaluable in identifying the preferred binding sites for a ligand.

Another approach involves screening relative binding affinities of a set of small molecules with a critical nucleic acid sequence. This is especially useful in searching for promising agents for inhibition of a TF-nucleic acid complex. This method is advantageous over other screening methods since the conditions are soft enough to preserve the complexes formed in solution yet versatile enough to provide a broad array of results for the interaction. Several other groups have explored the ability to probe DNA-ligand complexes through the use of multiple small molecules with a DNA sequence. Gabelica and co-workers have contributed substantially to this field and helped to pioneer the investigation of DNA complexes through ESI-MS. Early ESI-MS competition studies involved the dodecamer crystalized by Dickerson and co-workers [82] with standard minor groove binding compounds [28]. In decreasing order of preferred binding, they were able to identify the relative binding affinities of netropsin, distamycin A, DAPI, Hoescht, and berenil to an AT rich sequence in dsDNA by comparing the competitive behavior of two compounds at a time. Casagrande and co-workers have also provided extensive insights into the competitive binding of small molecules with quadruplex DNA. Calf thymus dsDNA was used as an internal competitor to help evaluate the selectivity of coronene and perylene compounds to quadruplex DNA [76].

In combination with modeling studies, interaction of a ligand with various DNA sequences—based on initial ESI-MS data—can aid in designing, synthetic binding agents with improved selectivity and specificity for DNA. Many results have shown that competition ESI-MS agrees well with data reported by other biophysical techniques, and several examples are described below. All results obtained using ESI-MS have been validated by alternative methods including, but not limited to, biosensor-SPR (biosensor-surface plasmon resonance), circular dichroism and fluorescence spectroscopy, DNase I footprinting and UV thermal melting.

### 5.1. Establishing a Competition Method Using Standard Minor Groove Binding Compounds

**Figure 2:**
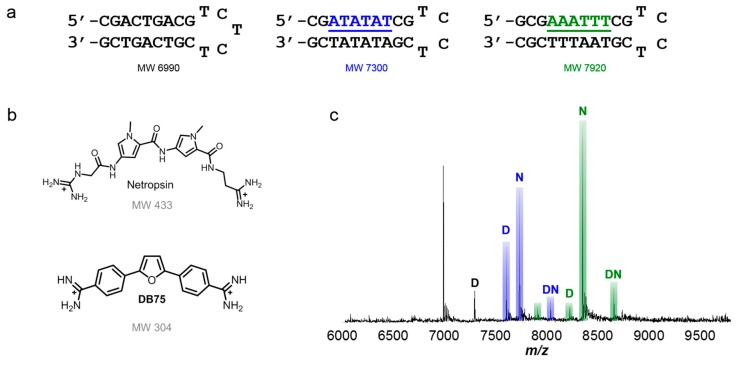
(**a**) Hairpin DNA sequences used for initial competition ESI-MS studies, including reference sequence; (**b**) Well-studied, classical, adenosine-thymidine (AT) rich, base pair-specific minor groove binding compounds netropsin and DB75; (**c**) spectrum of netropsin and DB75 competing for ATATAT and AAATTT sequence binding sites. Unbound DNA is unlabeled with corresponding color bar. Respective complexes labeled with “N” for netropsin and “D” for DB75. Complexes with both minor groove binding compounds are listed as “DN” for each titration. Ratio shown is a molar concentration ratio of [2 to 1]. T = thymidine; A = adenosine; G = guanidine; C = cytidine.

Analysis of competitive binding of compounds with a set of multiple, mixed sequence DNA sequences by ESI-MS is an attractive method that has been used in a number of applications. Initial tests were performed with minor groove binding compounds, netropsin [83,84,85,86,87,88] and DB75 [89,90,91], which are well-characterized. They are often used as standards in method development with DNA and minor groove binding compounds [92,93]. The compounds, as with many common minor groove binders, bind specifically to AT sequences. For simplicity, each DNA is denoted by its target site found in the sequence. For instance, if the target site in the sequence shown in Figure 2a is AAATTT, it is referred to as AAATTT. Additionally, a target site, such as AAATTT, is highlighted with a particular color font, in this case as green, and its corresponding peaks (*i.e.*, unbound AAATTT and/or AAATTT-ligand complexes) are also color-coded in green.

Titrations of A-tract and alternating AT sequences that have AAATTT and ATATAT binding sites in hairpin duplexes (Figure 2a) are shown with netropsin and DB75 in Figure 2b. At low molar concentrations of ligand to DNA, the interactions of a compound with its preferred binding site are detectable. For instance, a 1:1 complex formed between netropsin and AAATTT showed a higher peak intensity compared to a 1:1 complex formed with AAATTT and DB75 and unbound AAATTT (Figure 2c). The differences in peak intensities clearly show that AAATTT is the preferred target site for netropsin over ATATAT and this agrees well with other biophysical studies [94]. A relatively weak complex formed between a reference sequence (*i.e.*, one which does not contain multiple, adjacent AT base pairs) and DB75 was also detected at *m*/*z* ≈ 7250 and can occur through intercalation of the compound at GC base pair sites. The preferred binding of netropsin to AAATTT continued as concentrations of both compounds were increased showing that DB75 is clearly a weaker binding agent than netropsin for both AT-rich sequences. No significant free DNA, with the exception of the reference sequence, was left upon reaching a [2:1] molar concentration ratio.

### 5.2. Applying the Method Using Dimer-Forming Minor Groove Binding Compounds

**Figure 3:**
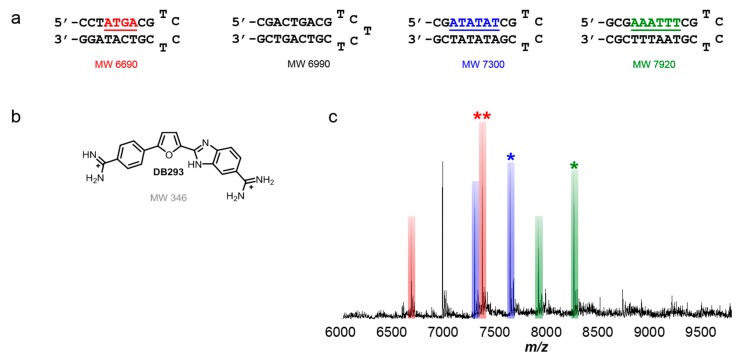
(**a**) Mixed hairpin DNA sequences used for ESI-MS studies, including a reference sequence; (**b**) ATGA-specific binding compound, DB293; (**c**) spectrum of DB293 with ATGA, ATATAT and AAATTT sequence binding sites. Unbound DNA remains unlabeled with corresponding color bar. Respective monomer complexes labeled with (*) for each titration. Dimer complexes (*i.e.*, 2 to 1 stoichiometry) labeled as (**). Ratio shown is a molar concentration ratio of [4 to 1]. T = thymidine; A = adenosine; G = guanidine; C = cytidine.

The minor groove binding compound, DB293, is an asymmetric dicationic diamidine known to recognize both AT-rich and mixed base pair DNA sequences (Figure 3a) [95,96,97,98]. It is a special compound in several ways, in that its binding mode varies depending on the sequence. DB293 (Figure 3b) is the first non-polyamide reported to bind as a dimer to a specific mixed site, GC-containing sequence while forming a monomer complex with AT-rich DNA sequences. Recognition of ATGA occurs through positive cooperative formation of a stacked dimer. Biosensor-SPR data has shown that the first DB293 molecule binds to ATGA, followed by a second molecule which binds with a higher binding constant than the first. NMR data has also shown that it binds in the minor groove as a dimer with an antiparallel stacking arrangement [95]. The interaction of DB293 with ATGA is clearly well-characterized and provides a good reference for a more complex system to study using ESI-MS.

A set of titrations was performed using DB293 with four different DNA sequences. DB293 was found to form a 1:1 complex with AT-rich sites but also formed a dimer complex with the mixed GC-containing sequence ATGA. At low molar concentrations, the compound was shown to bind well with the AT-rich sequences but no formation of a complex with ATGA was observed. However, as the concentration of DB293 was increased, the formation of a 2:1 complex of compound to ATGA became apparent which validates the positive, cooperative binding mode of DB293 since no 1:1 species was detected. The tallest peak observed corresponded to the DB293-ATGA complex and illustrated the preferred binding of DB293 with ATGA over interactions with AT-rich sequences as a 1:1 complex (see Figure 3c). The results found using ESI-MS agree well with other biophysical methods in both the relative binding affinities and binding modes of several well-studied DNA-small molecule interactions [96,97]. This study highlighted the ease of using several DNA sequences to simultaneously examine multiple interactions using a mixed set of DNA sequences and/or compounds. It also promoted competition among the ligands and DNAs which was determined by direct comparison of the peak intensities for the complexes and unbound DNAs.

#### 5.2.1. DNA and Analogs of DB293

**Figure 4:**
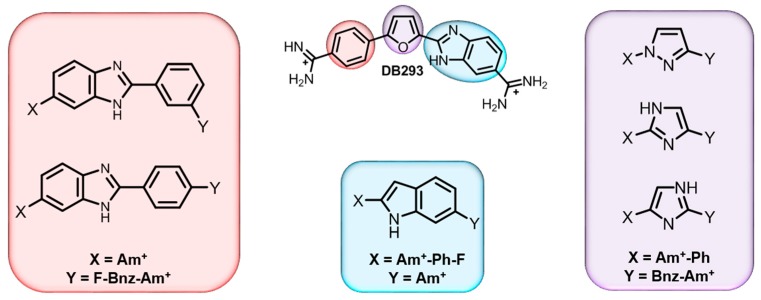
Functional group modifications to the DB293 parent compound. Elongation of the phenyl (Ph) binding site by inclusion of a benzimidazole (Bnz) motif, highlighted in red. Substitution of the benzimidazole (Bnz) group for indole, shown blue. To the right in purple, substitution of the central furan (F) group for azole moieties: pyrrazole and imidazole. Diamidine (Am*^+^*) functional groups were maintained for all groups. Ph = phenyl; Bnz = benzimidazole; F = furan; Am^+^ = amidine.

**Figure 5:**
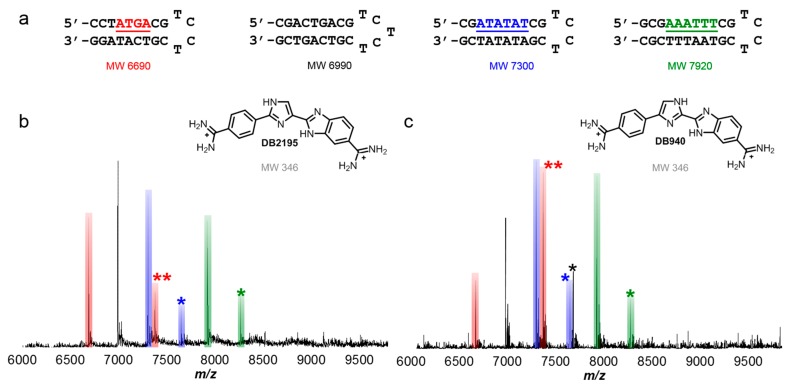
(**a**) Mixed hairpin DNA sequences used for ESI-MS studies, including a reference sequence; (**b**) spectrum of ATGA, ATATAT and AAATTT sequence binding sites with the DB293 analog, DB2195; (**c**) spectrum of ATGA, ATATAT and AAATTT sequences with the analog DB940. Unbound DNA remains unlabeled with corresponding color bar. Respective monomer complexes labeled with (*) for each titration. Dimer complexes (*i.e.*, 2 to 1 stoichiometry) labeled as (**). Molar concentration ratios for compound to DNA are [4 to 1]. T = thymidine; A = adenosine; G = guanidine; C = cytidine.

Competition ESI-MS was used to screen for relative binding of multiple compounds with a set of DNA sequences. The first set of studies followed the same approach where multiple DNA sequences and a single compound are screened for binding. Due to the extensive knowledge of DB293, it was used as a reference for the interaction of several DB293 analogs with a mixture of AT-rich and mixed-base pair sequences [71,80]. The set of titration experiments performed showed functional group modifications and structural variations influence minor groove binding (Figure 4). For instance, elongating the shape of DB293 inhibits dimer formation with the ATGA sequence and/or binding altogether. Slightly modifying the central five-membered ring through rearrangement and replacement of key hydrogen bond donor/acceptors also influences dimer formation of the compound with ATGA. The striking similarity among many of the structures, as shown in Figure 4 and Figure 5, would have suggested that their interactions with ATGA would be the very similar; however, significant differences in the binding modes were found with the ESI-MS method. Interestingly, the method also identified a new binding site for the dimer formation of one of the DB293 analogs with the reference sequence, CTGA, as denoted with (*) in Figure 5c. This sequence was initially used as a control or internal standard since it was believed to have no binding sites. These results raised two critical questions: (1) How did a seemingly simple reconfiguration of the central imidazole have such a large effect on the cooperative binding of DB2195 and DB940 with the ATGA target site and (2) how did it allow DB940 to cooperatively bind with the CTGA sequence? Using *ab initio* calculations, optimized structures of the isomers suggest the less cooperative DB2195 has a 20° twist in the phenyl-imidazole torsional angle. On the other hand, the placement of the imidazole nitrogens near the benzimidazole in DB940 allows for a planar structure, similar to DB293, and inherently allows better stacking in the minor groove. Without ESI-MS, this serendipitous discovery would not have been made. It provided insight into recognition of a more mixed-sequence site and inspired a new competition ESI-MS approach: screening for interaction of a compound with DNA having a known target site along with a family of mutated target site sequences.

#### 5.2.2. DB293 with ATGA and Mutant ATGA Sequences

A second, more sophisticated approach examines the competitive binding of a single compound with a DNA having a known target site against multiple, mutated target sites. The mutational selection of sequences modified in this way allows a focused approach to determining the optimum recognition site for any compound with DNA. Discovery of the new CTGA binding site mentioned above illustrates the power of using ESI-MS to examine DNA small molecule interactions focused around a specific sequence. The second approach was adopted to investigate sequence-specific binding of a compound with DNA sequence variants and to determine cooperative interactions. This assay used a known target site along with a set of mutated DNA binding sites, in addition to a pure GC sequence as the reference sequence. It was determined that the consensus ATGA sequence was the optimal recognition site for DB293 and agreed with earlier DNase I footprinting and other biophysical methods which identified ATGA as the target site [95].

**Figure 6:**
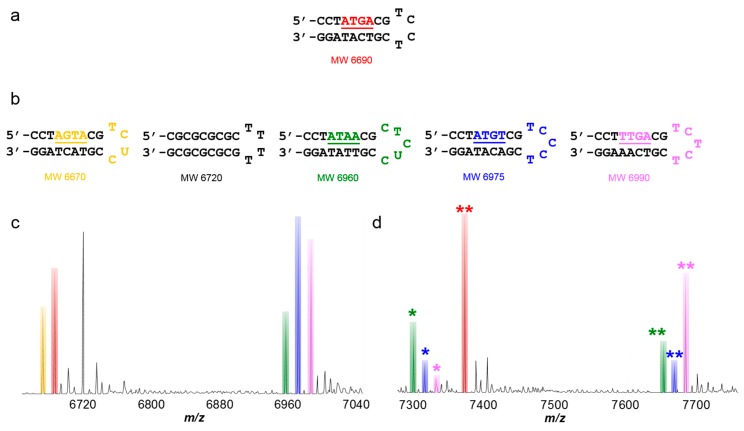
(**a**) The ATGA target base pair site for DB293; (**b**) ATGA-variant hairpin DNA sequences used for ESI-MS studies with preserved flanking base pair sequences, modified hairpin loops, and mutant target sites; (**c**) spectrum of unbound AGTA, ATGA, reference sequence, ATAA, ATGT and TTGA sequences; (**d**) spectrum of complexes formed between DB293 and ATAA, ATGT, TTGA and ATGA sequences. Unbound DNA is unlabeled with corresponding color bar. Respective monomer complexes labeled with (*) for each titration. Dimer complexes (*i.e.*, 2 to 1 stoichiometry) labeled as (**). Molar concentration ratios for compound to DNA are [4 to 1]. T = thymidine; A = adenosine; G = guanidine; C = cytidine.

A comparison of the consensus site with sequence variants offered insight into the effects the DNA sequence has on small molecule recognition (Figure 6). The importance of the TG critical sequence to binding is illustrated by the lack of complex formation with AGTA (yellow). The other sequences all formed both monomer and dimer complexes indicating lower positive cooperativity then with ATGA where no monomer binding is observed. Interestingly, the TTGA (pink) sequence has high positive cooperativity with little monomer binding while the sequence isomer, ATGT (blue), has little positive cooperativity. Because of this study, the importance of (i) functional group effects on minor groove recognition; (ii) identification of sequence specificity for a ligand with an optimal binding site; and (iii) the influence of DNA sequence on binding cooperativity were highlighted. The components which make DB293 ideal for recognition with ATGA were pinpointed. Additionally, the study provided a better understanding of how critical the sequence is even when the base pair composition is maintained, offering a new paradigm for DNA minor groove recognition.

### 5.3. Using Competition to Study Complex, Mixed-Site Sequences with Minor Groove Binding Compounds

A fairly recent study, which also incorporated competition ESI-MS, examined recognition of longer and more specific minor groove target sites. These sites were expanded to include two, separate AT-rich sites flanking one or two GC base pairs. Since many minor groove binding compounds are specific for pure AT base pair containing sequences, this study represented a breakthrough in the development of more specific DNA recognizing compounds. This approach is important for therapeutic potential since increasing specificity should yield a concomitant decrease in toxicity or other side effects. DNA sequences were simultaneously screened with a small molecule to create an ideal competitive binding environment. Each sample contained a 100% GC base pair rich sequence (*i.e.*, no AT base pairs) as an internal standard for better comparison of peak intensities. Molecular weights of the DNAs were adjusted so differences in the weights arose from substitution of an inosine for guanine (removing the C2 amino) in addition to modification of the hairpin loop.

**Figure 7:**
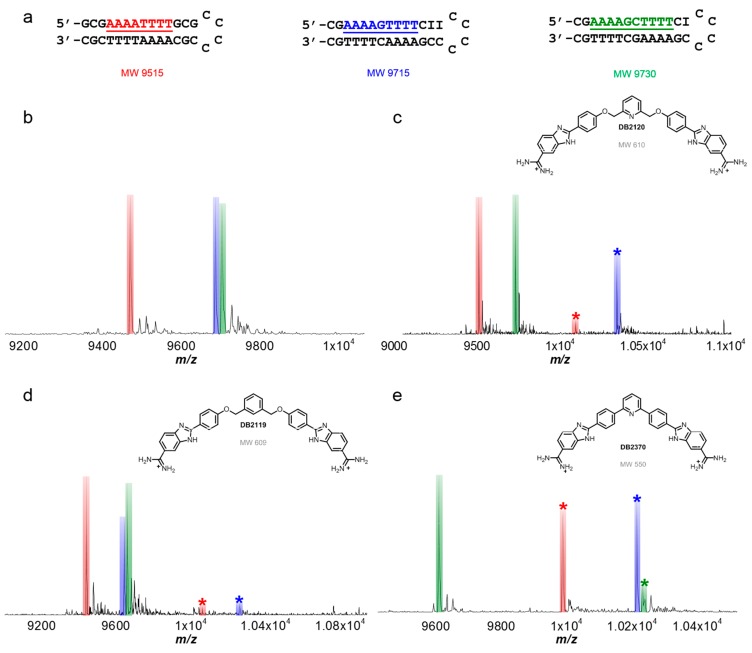
(**a**) Mixed hairpin DNA sequences used for ESI-MS studies; (**b**) spectrum of A4T4 and mixed sequences A4GT4 and A4GCT4 only; (**c**) spectrum of A4T4, A4GT4 and A4GCT4 sequences with DB2120; (**d**) spectrum of DNA sequences and DB2119; (**e**) spectrum of A4T4, A4GT4 and A4GCT4 sequences with DB2370. Unbound DNA remains unlabeled with corresponding color bar. Respective complexes labeled with (*) for each titration. Molar concentration ratios for compound to DNA are [4 to 1]. T = thymidine; A = adenosine; G = guanidine; C = cytidine.

The dicationic diamidine DB2120 (Figure 7) is a compound with a symmetric structure which contains two motifs known to binding AT-rich sites separated by a central pyridyl for potential G-base binding. Upon titrating in DB2120, all free A4GT4 was bound by the compound as a 1:1 complex. Only weak 1:1 complexes were formed between DB2120 with the DNA sequences containing none or two GC base pairs (*i.e.*, A4T4 and A4GCT4) which clearly indicates the strong and very selective binding for the A4GT4 sequence (Figure 7). This is the first example of design of a minor groove binding agent for mixed base pair sequence recognition and ESI-MS was a key component in establishing the recognition sequence. Two analogs of DB2120 were next investigated, DB2119 and DB2370 (Figure 7). At a [4:1] molar concentration ratio of ligand to DNA, both analogs were found to interact weakly with the two GC base pair sequence (A4GCT4). Each analog formed a complex with A4T4 and A4GT4 sequences of similar relative intensities, which indicate neither DB2119 nor DB2370 can discriminate against zero or one GC base pair in the target site. The structure of DB2119 lacks a hydrogen bond acceptor, such as the pyridine nitrogen in DB2120, whereas DB2370 lacks the alkyl linker giving it a more rigid structure. The results obtained by ESI-MS for both analogs agree very well with biosensor-SPR, circular dichroism, modeling, and thermal melting studies [81].

## 6. Comparison of Mass Analyzers and Ionization Methods

Analysis of whole or intact systems (e.g., DNA-complexes, apo- *vs.* holo- proteins, *etc.*) are critical to understanding the structural and functional properties of many biological systems. This becomes extremely important for analysis of low molecular weight binding compounds and during native MS of biomolecular systems. Considerations in the application of MS to study DNA-small molecule interactions include the type of instrument used for analysis. Hybrid quadrupole-time-of-flight (Q-ToF) mass analyzers are among the most widely used in mass spectrometry. The resolution, or resolving power, of a mass analyzer is characterized by its ability to distinguish neighboring ion peaks. The better the peak separation, the higher the resolution. The resolving power of time-of-flight (ToF) mass spectrometers are inherently poorer in comparison to quadrupole and orbitrap mass spectrometers, and while quadruple mass analyzers offer better resolution than time-of-flight, the resolving power of an orbitrap typically exceeds a hybrid Q-ToF mass spectrometer.

**Figure 8:**
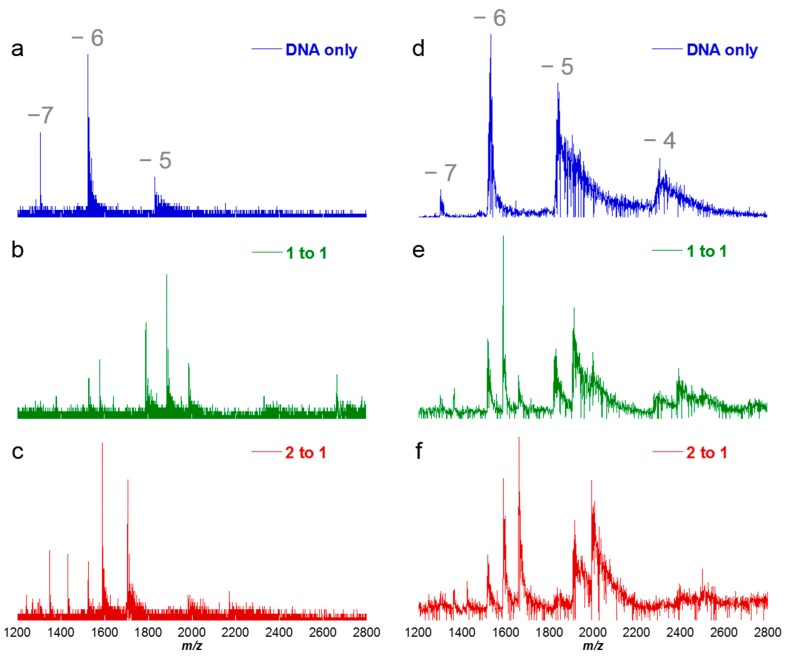
Comparison of spectra of DNA and complexes obtained using different mass analyzers. Peaks shown correspond to molar concentration ratios of [0:1], [1:1] and [2:1] for netropsin to DNA. Several negative charge states are shown ranging from −7 to −4. Stoichiometric ratios of 1:1 and 2:1 binding for netropsin to DNA are observed at the corresponding charge states. (**a**–**c**) Left column figures are for Q-ToF mass analyzer and (**d**–**f**) right column figures for orbitrap mass analyzer. Figures shown in blue (**a**,**d**) indicate DNA only in the sample mixture (no added netropsin). Those shown as green (**b**,**e**) are for molar concentration ratios of 1 to 1 (netropsin to DNA) and figures in red (**c**,**f**) correlate to molar concentration ratios of 2 to 1 (2 netropsin to 1 DNA).

For many DNA-small molecule studies, an ESI source with a hybrid Q-ToF mass analyzer gives adequate resolution for the system under study as well as reproducible results. In addition to ESI-Q-ToF, mass spectrometers with an ESI source and an orbitrap mass analyzer are becoming increasingly popular in the analysis of biomacromolecules. Both types of ESI mass spectrometers are powerful in the analysis of large biomolecules and are capable of scanning high molecular weight systems. An example comparing the results obtained, using two instruments containing an ESI source and outfitted with different mass analyzers, is shown below (Figure 8) and discussed in the following sections.

**Figure 9:**
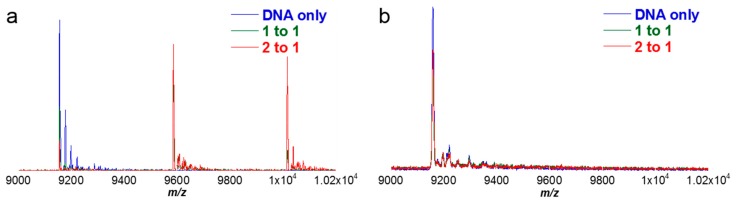
Spectra of DNA-netropsin complexes analyzed by different ionization techniques. Peaks from the [0:1], [1:1] and [2:1] molar concentration ratios of netropsin to DNA were overlaid for simple comparison. (**a**) Deconvoluted spectrum of DNA and complexes ionized through electrospray and (**b**) full MALDI spectrum showing no detectable netropsin-DNA complexes at any ratio.

In addition to ESI mass spectrometers, matrix assisted laser desorption ionization (MALDI) is a key technique in the analyses of biological systems. MALDI-MS begins by mixing the sample with a high proportion of matrix solution followed by spotting of the sample onto a specialized plate. After the solvent solution evaporates, the sample plate is inserted into the instrument for analysis. MALDI differs from ESI ionization in that radiation is used to remove the samples and matrix while simultaneously ionizing the molecules. Combined with a time-of-flight mass analyzer, MALDI has become a powerful tool in the analysis of biomacromolecules having molecular weights over 200 kDa and with high sensitivity [68]. Due to the high scanning capability and common practice in bioanalytical studies, an example of DNA-small molecule complexes analyzed using a MALDI-ToF mass spectrometer are shown in the following sections (Figure 9) to compare MALDI and ESI ionization sources.

### 6.1. ESI-Q-ToF MS

In Figure 8a, the sample containing DNA only (no netropsin) was labeled as “DNA only” and identified with blue peaks. The titration sample having equal molar ratios of netropsin to AAAAGCTTTT was labeled as “[1:1]” and shown with green peaks (Figure 8b). The final sample doubled the amount of netropsin to AAAAGCTTTT, bringing the ratio to [2:1] and labeled accordingly with red peaks (Figure 8c). Since there are two short AT-rich binding sites on the DNA (5′-AAAAGCTTTT-3′ and 3′-TTTTCGAAAA-5′) of the size required for netropsin, one would expect to find 1:1 complexes at low concentrations of netropsin and 2:1 complexes at high netropsin concentrations. The tallest peak intensity in Figure 8a corresponds to DNA having a −6 charge to give *m*/*z* 1525 (e.g., 9156 − 6 = 9150 ÷ 6 = 1525). The overall charge states of the DNA did not change when netropsin was added, so a −6 peak for a 1:1 binding complex of netrospin-DNA would be found near *m*/*z* 1595. A charge state of −6 was not the only charge present for the system. Net charges of −5 and −7 were also detected in the spectrum, but with less intensity compared to −6, regardless if the DNA was bound or not. From this set of spectra, it is clear netropsin binds more strongly as a 1:1 stoichiometric complex to AAAAGCTTTT and goes to 2:1 as the concentration of netropsin is increased.

### 6.2. ESI-Orbitrap MS

The third spectrum, Figure 8d, was analyzed by ESI using an orbitrap mass analyzer. An orbitrap mass analyzer can offer more features than the Q-ToF, such as higher mass resolution, accurate mass, a higher scanning range (*m*/*z* up to 6000 *vs.* 4000) [68] and less sample consumption. With this instrument, multiple NH_4_^+^ adducts with the DNA were found which lowered the sensitivity for any specific component, whereas nominal amounts of adducts were detected using the Q-ToF. A similar problem was reported by Balthasart and co-workers with quadruplex DNA which sometimes traps a cation in the diagonal loop [99]. While hairpin duplex DNAs do not contain the same loops found in quadruplex structures, it is possible there may be an NH_4_^+^ cation contained within the DNA hairpin loop. A possible explanation for this is that the conditions used during Q-ToF analyses may be harsh enough to remove an ammonium ion compared to the orbitrap mass analyzer, so non-specifically bound NH_4_^+^ cations in the hairpin loop are lost during the Q-ToF analysis. Interestingly, the more abundant charge state detected by the orbitrap mass analyzer corresponded to the −6 charged species *vs.* −5 once netropsin was added with the Q-ToF.

### 6.3. MALDI-ToF

MALDI is useful for many reasons, including a scan range of up to 200,000 Da and can vaporize samples with relative ease. Samples having identical molar ratios of netropsin to AAAAGCTTTT were scanned. A deconvoluted spectrum of the samples analyzed using ESI-Q-ToF (Figure 9a) is compared with samples analyzed by MALDI-ToF (Figure 9b). Unfortunately, issues exist such as in order to retrieve and ionize the DNA complex, a laser with high intensity must be used to desorb the complex from the matrix surface. In doing so, the energy to desorb the analyte is often greater than the energy between the DNA and non-covalently bound small molecule. Similar results were observed when running MALDI to analyze samples of netropsin and AAAAGCTTTT and find that even with the lowest intensity possible, the energy is greater than that needed to form the complex and only free DNA is detected, even at the highest concentration of netropsin.

There are other concerns in using MALDI to study DNA-small molecule interactions. For example, positive ion mode is intrinsically more sensitive in MS analyses but in MALDI-MS, nucleic acids often need to be in positive mode which poses a problem for high negatively charged species, such as DNA. Another issue is separation of the matrix and DNA-complexes during the drying process and crystallization of the matrix which creates heterogeneity in the sample. The “sweet spot”—a favorable, yet unknown formation of crystals containing the analyte—must be determined, but only after the loading plate has been inserted into the instrument. Additionally, it is also possible interference with the DNA-small molecule complex occurs from the matrix and can cause the compound to dissociate from the DNA. The conditions used in MALDI are considerably harsher than those used in ESI and are particularly important when studying non-covalent interactions such as small molecule-DNA complexes. This is likely the reason for the disappearance of any netropsin-DNA complex peaks when using MALDI compared to analysis by ESI-Q-ToF and ESI-orbitrap mass spectrometers.

The results obtained using an ESI source are consistent unlike those by MALDI. Both the ESI-Q-ToF and ESI-orbitrap instruments indicate the majority of DNA was bound to netropsin by [2:1] ratios with both 1:1 and 2:1 stoichiometric complexes. Riccardi-Sirtori and co-workers have shown similar findings in the analysis of DNA-small molecule complexes using both ESI-Q-ToF and ESI-orbitrap mass spectrometers [100]. Their results were comparable to each other based on the apparent binding constants determined using both instruments types and agree well with the results shown in Figure 8. Slight variability can exist in the relative peak intensities when using different mass analyzers. For instance, instrument parameters were optimized according to each instrument and the conditions necessary for ESI-orbitrap and ESI-Q-ToF mass spectrometers vary. Considering that there are differences even in identical instruments, many factors, even seemingly minor ones such as when the instrument was last tuned, calibration, even the time of day, can affect instrument performance. These factors should be considered in comparative studies of macromolecular systems but can be simplified when using competition ESI-MS.

## 7. Conclusions

ESI-MS has already been established as a powerful tool in the characterization of small molecules and biomacromolecular systems. Development of a competition ESI-MS method has allowed ESI-MS to be used as a high throughput screening technique to simultaneously investigate the binding interactions of minor groove binding compounds with multiple DNA sequences. Important information such as stoichiometry, relative binding affinities, cooperativity, and binding modes are easily determined with competition ESI-MS. Sample preparation is simple and analyses are quick which becomes especially important for sensitive systems including DNA-small molecule, DNA-protein, protein-protein, DNA-RNA hybrids, and protein-ligand complexes. This method is especially attractive since theoretically any number of interactions can be examined, as long as the molecular weights are distinguishable for both the free/unbound species and complexed systems. Additionally, inclusion of a reference or control system within the sample with which to compare system(s) of interest can allow the relative binding affinities to be determined. The results obtained by competition ESI-MS experiments offer clear spectral comparisons and reproducible results that are in agreement with other biophysical methods.

## References

[1] Lightwahl K.J., Springer D.L., Winger B.E., Edmonds C.G., Camp D.G., Thrall B.D., Smith R.D. (1993). Observation of a small oligonucleotide duplex by electrospray ionization mass-spectrometry. J. Am. Chem. Soc..

[2] Gabelica V., Vreuls C., Filee P., Duval V., Joris B., de Pauw E. (2002). Advantages and drawbacks of nanospray for studying noncovalent protein-DNA complexes by mass spectrometry. Rapid Commun. Mass Spectrom..

[3] Gidden J., Ferzoco A., Baker E.S., Bowers M.T. (2004). Duplex formation and the onset of helicity in poly d(CG)(n) oligonucleotides in a solvent-free environment. J. Am. Chem. Soc..

[4] Madsen J.A., Brodbelt J.S. (2010). Asymmetric charge partitioning upon dissociation of DNA duplexes. J. Am. Soc. Mass Spectrom..

[5] Wan K.X., Gross M.L., Shibue T. (2000). Gas-phase stability of double-stranded oligodeoxynucleotides and their noncovalent complexes with DNA-binding drugs as revealed by collisional activation in an ion trap. J. Am. Soc. Mass Spectrom..

[6] Barylyuk K., Gulbakan B., Xie X.S., Zenobi R. (2013). DNA oligonucleotides: A model system with tunable binding strength to study monomer-dimer equilibria with electrospray ionization-mass spectrometry. Anal. Chem..

[7] Marchand A., Ferreira R., Tateishi-Karimata H., Miyoshi D., Sugimoto N., Gabelica V. (2013). Sequence and solvent effects on telomeric DNA bimolecular G-quadruplex folding kinetics. J. Phys. Chem. B.

[8] Ferreira R., Marchand A., Gabelica V. (2012). Mass spectrometry and ion mobility spectrometry of G-quadruplexes. A study of solvent effects on dimer formation and structural transitions in the telomeric DNA sequence d(TAGGGTTAGGGT). Methods.

[9] Bayer E., Bauer T., Schmeer K., Bleicher K., Maier M., Gaus H.J. (1994). Analysis of double-stranded oligonucleotides by electrospray mass spectrometry. Anal. Chem..

[10] Loo J.A. (1997). Studying noncovalent protein complexes by electrospray ionization mass spectrometry. Mass Spectrom. Rev..

[11] Hofstadler S.A., Griffey R.H. (2001). Analysis of noncovalent complexes of DNA and RNA by mass spectrometry. Chem. Rev..

[12] Hoyne P.R., Benson L.M., Veenstra T.D., Maher L.J., Naylor S. (2001). RNA-RNA noncovalent interactions investigated by microspray ionization mass spectrometry. Rapid Commun. Mass Spectrom..

[13] Ni J.S., Chan K. (2001). Sequence verification of oligonucleotides by electrospray quadrupole time-of flight mass spectrometry. Rapid Commun. Mass Spectrom..

[14] Fabris D. (2011). MS analysis of nucleic acids in the post-genomic era. Anal. Chem..

[15] Gale D.C., Smith R.D. (1995). Characterization of noncovalent complexes formed between minor groove binding molecules and duplex DNA by electrospray ionization mass spectrometry. J. Am. Soc. Mass Spectrom..

[16] Chiang C.K., Lin Y.W., Hu C.C., Chang H.T. (2009). Using electrospray ionization mass spectrometry to explore the interactions among polythymine oligonucleotides, ethidium bromide, and mercury ions. J. Am. Soc. Mass Spectrom..

[17] Talib J., Green C., Davis K.J., Urathamakul T., Beck J.L., Aldrich-Wright J.R., Ralph S.F. (2008). A comparison of the binding of metal complexes to duplex and quadruplex DNA. Dalton Trans..

[18] Wu Q.Y., Cheng X.H., Hofstadler S.A., Smith R.D. (1996). Specific metal-oligonucleotide binding studied by high resolution tandem mass spectrometry. J. Mass Spectrom..

[19] Wang Z., Cui M., Song F., Lu L., Liu Z., Liu S. (2005). Evaluation of flavonoids binding to DNA duplexes by electrospray ionization mass spectrometry. J. Am. Soc. Mass Spectrom..

[20] Wan K., Shibue T., Gross M.L. (2000). Non-covalent complexes between DNA-binding drugs and double-stranded oligonucleotides: A study by ESI ion-trap mass spectrometry. J. Am. Chem. Soc..

[21] Doria F., Nadai M., Folini M., Scalabrin M., Germani L., Sattin G., Mella M., Palumbo M., Zaffaroni N., Fabris D. (2013). Targeting loop adenines in G-quadruplex by a selective oxirane. Chemistry.

[22] Urathamakul T., Beck J.L., Sheil M.M., Aldrich-Wright J.R., Ralph S.F. (2004). A mass spectrometric investigation of non-covalent interactions between ruthenium complexes and DNA. Dalton Trans..

[23] Potier N., Donald L.J., Chernushevich I., Ayed A., Ens W., Arrowsmith C.H., Standing K.G., Duckworth H.W. (1998). Study of a noncovalent trp repressor: DNA operator complex by electrospray ionization time-of-flight mass spectrometry. Protein Sci..

[24] Gale D.C., Goodlett D.R., Lightwahl K.J., Smith R.D. (1994). Observation of duplex DNA-drug noncovalent complexes by electrospray-ionization mass-spectrometry. J. Am. Chem. Soc..

[25] Pelton J.G., Wemmer D.E. (1988). Structural modeling of the distamycin A-d(CGCGAATTCGCG)2 complex using 2D NMR and molecular mechanics. Biochemistry.

[26] Pelton J.G., Wemmer D.E. (1989). Structural characterization of a 2:1 distamycin A·d(CGCAAATTGGC) complex by two-dimensional NMR. Proc. Natl. Acad. Sci. USA.

[27] Pelton J.G., Wemmer D.E. (1990). Structure and dynamics of distamycin a with d(CGCAAATTGGC):D(GCCAATTTGCG) at low drug:DNA ratios. J. Biomol. Struct. Dyn..

[28] Gabelica V., de Pauw E., Rosu F. (1999). Interaction between antitumor drugs and a double-stranded oligonucleotide studied by electrospray ionization mass spectrometry. J. Mass Spectrom..

[29] Greig M.J., Robinson J.M. (2000). Detection of oligonucleotide-ligand complexes by ESI-MS (DOLCE-MS) as a component of high throughput screening. J. Biomol. Screen..

[30] Rosu F., de Pauw E., Guittat L., Alberti P., Lacroix L., Mailliet P., Riou J.F., Mergny J.L. (2003). Selective interaction of ethidium derivatives with quadruplexes: An equilibrium dialysis and electrospray ionization mass spectrometry analysis. Biochemistry.

[31] Beck J.L., Gupta R., Urathamakul T., Williamson N.L., Sheil M.M., Aldrich-Wright J.R., Ralph S.F. (2003). Probing DNA selectivity of ruthenium metallointercalators using ESI mass spectrometry. Chem. Commun..

[32] Mazzitelli C.L., Chu Y., Reczek J.J., Iverson B.L., Brodbelt J.S. (2007). Screening of threading bis-intercalators binding to duplex DNA by electrospray ionization tandem mass spectrometry. J. Am. Soc. Mass Spectrom..

[33] Bahr M., Gabelica V., Granzhan A., Teulade-Fichou M.P., Weinhold E. (2008). Selective recognition of pyrimidine-pyrimidine DNA mismatches by distance-constrained macrocyclic bis-intercalators. Nucleic Acids Res..

[34] Gupta R., Beck J.L., Ralph S.F., Sheil M.M., Aldrich-Wright J.R. (2004). Comparison of the binding stoichiometries of positively charged DNA-binding drugs using positive and negative ion electrospray ionization mass spectrometry. J. Am. Soc. Mass Spectrom..

[35] Wortmann A., Jecklin M.C., Touboul D., Badertscher M., Zenobi R. (2008). Binding constant determination of high-affinity protein-ligand complexes by electrospray ionization mass spectrometry and ligand competition. J. Mass Spectrom..

[36] Cheng X.H., Chen R.D., Bruce J.E., Schwartz B.L., Anderson G.A., Hofstadler S.A., Gale D.C., Smith R.D., Gao J.M., Sigal G.B. (1995). Using electrospray-ionization FTICR mass-spectrometry to study competitive-binding of inhibitors to carbonic-anhydrase. J. Am. Chem. Soc..

[37] Casini A., Gabbiani C., Michelucci E., Pieraccini G., Moneti G., Dyson P.J., Messori L. (2009). Exploring metallodrug-protein interactions by mass spectrometry: Comparisons between platinum coordination complexes and an organometallic ruthenium compound. J. Biol. Inorg. Chem..

[38] Krishnaswamy S.R., Williams E.R., Kirsch J.F. (2006). Free energies of protein-protein association determined by electrospray ionization mass spectrometry correlate accurately with values obtained by solution methods. Protein Sci..

[39] Jorgensen T.J.D., Roepstorff P., Heck A.J.R. (1998). Direct determination of solution binding constants for noncovalent complexes between bacterial cell wall peptide analogues and vancomycin group antibiotics by electrospray ionization mass spectrometry. Anal. Chem..

[40] Jorgensen T.J.D., Staroske T., Roepstorff P., Williams D.H., Heck A.J.R. (1999). Subtle differences in molecular recognition between modified glycopeptide antibiotics and bacterial receptor peptides identified by electrospray ionization mass spectrometry. J. Chem. Soc. Perkin Trans. 2.

[41] Birrento M.L., Bryan T.M., Samosorn S., Beck J.L. (2015). ESI-MS investigation of an equilibrium between a bimolecular quadruplex DNA and a duplex DNA/RNA hybrid. J. Am. Soc. Mass Spectrom..

[42] Malgowska M., Gudanis D., Kierzek R., Wyszko E., Gabelica V., Gdaniec Z. (2014). Distinctive structural motifs of RNA G-quadruplexes composed of AGG, CGG and UGG trinucleotide repeats. Nucleic Acids Res..

[43] Bai L.P., Liu J., Han L., Ho H.M., Wang R., Jiang Z.H. (2014). Mass spectrometric studies on effects of counter ions of TMPyP4 on binding to human telomeric DNA and RNA G-quadruplexes. Anal. Bioanal. Chem..

[44] Stephenson W., Asare-Okai P.N., Chen A.A., Keller S., Santiago R., Tenenbaum S.A., Garcia A.E., Fabris D., Li P.T. (2013). The essential role of stacking adenines in a two-base-pair RNA kissing complex. J. Am. Chem. Soc..

[45] Tan W., Yuan G. (2013). Electrospray ionization mass spectrometric exploration of the high-affinity binding of three natural alkaloids with the mRNA G-quadruplex in the BCL2 5′-untranslated region. Rapid Commun. Mass Spectrom..

[46] Cui X., Lin S., Zhou J., Yuan G. (2012). Investigation of non-covalent interaction of natural flexible cyclic molecules with telomeric RNA G-quadruplexes by electrospray ionization mass spectrometry. Rapid Commun. Mass Spectrom..

[47] Asare-Okai P.N., Chow C.S. (2011). A modified fluorescent intercalator displacement assay for RNA ligand discovery. Anal. Biochem..

[48] Collie G.W., Parkinson G.N., Neidle S., Rosu F., de Pauw E., Gabelica V. (2010). Electrospray mass spectrometry of telomeric RNA (TERRA) reveals the formation of stable multimeric G-quadruplex structures. J. Am. Chem. Soc..

[49] Akashi S., Watanabe M., Heddle J.G., Unzai S., Park S.Y., Tame J.R. (2009). RNA and protein complexes of TRP RNA-binding attenuation protein characterized by mass spectrometry. Anal. Chem..

[50] Kieltyka J.W., Chow C.S. (2006). Probing RNA hairpins with cobalt(III)hexammine and electrospray ionization mass spectrometry. J. Am. Soc. Mass Spectrom..

[51] Sannes-Lowery K.A., Griffey R.H., Hofstadler S.A. (2000). Measuring dissociation constants of RNA and aminoglycoside antibiotics by electrospray ionization mass spectrometry. Anal. Biochem..

[52] Amato J., Oliviero G., de Pauw E., Gabelica V. (2009). Hybridization of short complementary PNAs to G-quadruplex forming oligonucleotides: An electrospray mass spectrometry study. Biopolymers.

[53] Paul A., Sengupta P., Krishnan Y., Ladame S. (2008). Combining G-quadruplex targeting motifs on a single peptide nucleic acid scaffold: A hybrid (3 + 1) PNA-DNA bimolecular quadruplex. Chemistry.

[54] Datta B., Bier M.E., Roy S., Armitage B.A. (2005). Quadruplex formation by a guanine-rich PNA oligomer. J. Am. Chem. Soc..

[55] Degnan B.M., Degnan S.M., Naganuma T., Morse D.E. (1993). The *ETS* multigene family is conserved throughout the metazoa. Nucleic Acids Res..

[56] Arvand A., Denny C.T. (2001). Biology of EWS/ETS fusions in Ewing's family tumors. Oncogene.

[57] Clark J.P., Cooper C.S. (2009). *ETS* gene fusions in prostate cancer. Nat. Rev. Urol..

[58] Wei G.H., Badis G., Berger M.F., Kivioja T., Palin K., Enge M., Bonke M., Jolma A., Varjosalo M., Gehrke A.R. (2010). Genome-wide analysis of ETS-family DNA-binding *in vitro* and *in vivo*. EMBO J..

[59] Zaret K.S., Carroll J.S. (2011). Pioneer transcription factors: Establishing competence for gene expression. Genes Dev..

[60] Hollenhorst P.C., McIntosh L.P., Graves B.J. (2011). Genomic and biochemical insights into the specificity of ETS transcription factors. Annu. Rev. Biochem..

[61] Wang Y., Feng L., Said M., Balderman S., Fayazi Z., Liu Y., Ghosh D., Gulick A.M. (2005). Analysis of the 2.0 A crystal structure of the protein-DNA complex of the human PDEF ETS domain bound to the prostate specific antigen regulatory site. Biochemistry.

[62] Babayeva N.D., Wilder P.J., Shiina M., Mino K., Desler M., Ogata K., Rizzino A., Tahirov T.H. (2010). Structural basis of ETS1 cooperative binding to palindromic sequences on stromelysin-1 promoter DNA. Cell Cycle.

[63] Cheng X., Morin P.E., Harms A.C., Bruce J.E., Ben-David Y., Smith R.D. (1996). Mass spectrometric characterization of sequence-specific complexes of DNA and transcription factor PU.1 DNA binding domain. Anal. Biochem..

[64] Darnell J.E. (2002). Transcription factors as targets for cancer therapy. Nat. Rev. Cancer.

[65] Koehler A.N. (2010). A complex task? Direct modulation of transcription factors with small molecules. Curr. Opin. Chem. Biol..

[66] Talib J., Beck J.L., Urathamakul T., Nguyen C.D., Aldrich-Wright J.R., Mackay J.P., Ralph S.F. (2009). A mass spectrometric investigation of the ability of metal complexes to modulate transcription factor activity. Chem. Commun..

[67] Gaskell S.J. (1997). Electrospray: Principles and practice. J. Mass Spectrom..

[68] Dass C. (2007). Fundamentals of Contemporary Mass Spectrometry.

[69] Beck J.L. (2011). Developments in electrospray ionization mass spectrometry of non-covalent DNA-ligand complexes. Aust. J. Chem..

[70] Rosu F., de Pauw E., Gabelica V. (2008). Electrospray mass spectrometry to study drug-nucleic acids interactions. Biochimie.

[71] Laughlin S., Wang S., Kumar A., Boykin D.W., Wilson W.D. (2014). A novel approach using electrospray ionization mass spectrometry to study competitive binding of small molecules with mixed DNA sequences. Anal. Bioanal. Chem..

[72] Guo X., Bruist M.F., Davis D.L., Bentzley C.M. (2005). Secondary structural characterization of oligonucleotide strands using electrospray ionization mass spectrometry. Nucleic Acids Res..

[73] Brodbelt J.S. (2010). Evaluation of DNA/ligand interactions by electrospray ionization mass spectrometry. Annu. Rev. Anal. Chem..

[74] Amato J., Olivier G., Borbone N., D’Errico S., Piccialli G., Mailliet P., Rosu F., de Pauw E., Gabelica V. (2008). Ligand binding to tetra-end-linked (TGGGGT)4 G-quadruplexes: An electrospray mass spectrometry study. Nucleic Acids Symp. Ser..

[75] Gabelica V., Galic N., Rosu F., Houssier C., De Pauw E. (2003). Influence of response factors on determining equilibrium association constants of non-covalent complexes by electrospray ionization mass spectrometry. J. Mass Spectrom..

[76] Casagrande V., Alvino A., Bianco A., Ortaggi G., Franceschin M. (2009). Study of binding affinity and selectivity of perylene and coronene derivatives towards duplex and quadruplex DNA by ESI-MS. J. Mass Spectrom..

[77] Kempen E.C., Brodbelt J.S. (2000). A method for the determination of binding constants by electrospray ionization mass spectrometry. Anal. Chem..

[78] Gabelica V., Rosu F., de Pauw E. (2009). A simple method to determine electrospray response factors of noncovalent complexes. Anal. Chem..

[79] Kapur A., Beck J.L., Sheil M.M. (1999). Observation of daunomycin and nogalamycin complexes with duplex DNA using electrospray ionisation mass spectrometry. Rapid Commun. Mass Spectrom..

[80] Laughlin S., Wang S., Kumar A., Farahat A.A., Boykin D.W., Wilson W.D. (2015). Resolution of mixed site DNA complexes with dimer-forming minor-groove binders by using electrospray ionization mass spectrometry: Compound structure and DNA sequence effects. Chemistry.

[81] Paul A., Nanjunda R., Kumar A., Laughlin S., Nhili R., Depauw S., Deuser S.S., Chai Y., Chaudhary A.S., David-Cordonnier M.H. (2015). Mixed up minor groove binders: Convincing A.T specific compounds to recognize a G.C base pair. Bioorg. Med. Chem. Lett..

[82] Wing R., Drew H., Takano T., Broka C., Tanaka S., Itakura K., Dickerson R.E. (1980). Crystal structure analysis of a complete turn of B-DNA. Nature.

[83] Wartell R.M., Larson J.E., Wells R.D. (1974). Netropsin. A specific probe for A-T regions of duplex deoxyribonucleic acid. J. Biol. Chem..

[84] Zimmer C., Luck G., Fric I. (1976). Duplex structure formation between oligo(dA)’s and oligo(dT)’s generated by thymine-specific interaction with netropsin. Nucleic Acids Res..

[85] Patel D.J., Canuel L.L. (1977). Netropsin-poly(dA-dT) complex in solution: Structure and dynamics of antibiotic-free base pair regions and those centered on bound netropsin. Proc. Natl. Acad. Sci. USA.

[86] Berman H.M., Neidle S., Zimmer C., Thrum H. (1979). Netropsin, a DNA-binding oligopeptide structural and binding studies. Biochim. Bbiophys. Acta.

[87] Zimmer C., Marck C., Schneider C., Guschlbauer W. (1979). Influence of nucleotide sequence on dA.dT-specific binding of netropsin to double stranded DNA. Nucleic Acids Res..

[88] Marky L.A., Breslauer K.J. (1987). Origins of netropsin binding affinity and specificity: Correlations of thermodynamic and structural data. Proc. Natl. Acad. Sci. USA.

[89] Zhao M., Ratmeyer L., Peloquin R.G., Yao S., Kumar A., Spychala J., Boykin D.W., Wilson W.D. (1995). Small changes in cationic substituents of diphenylfuran derivatives have major effects on the binding affinity and the binding mode with RNA helical duplexes. Bioorg. Med. Chem..

[90] Laughton C.A., Tanious F., Nunn C.M., Boykin D.W., Wilson W.D., Neidle S. (1996). A crystallographic and spectroscopic study of the complex between d(CGCGAATTCGCG)2 and 2,5-*bis*(4-guanylphenyl)furan, an analogue of berenil. Structural origins of enhanced DNA-binding affinity. Biochemistry.

[91] Bailly C., Dassonneville L., Carrasco C., Lucas D., Kumar A., Boykin D.W., Wilson W.D. (1999). Relationships between topoisomerase II inhibition, sequence-specificity and DNA binding mode of dicationic diphenylfuran derivatives. Anticancer Drug Des..

[92] Boger D.L., Fink B.E., Brunette S.R., Tse W.C., Hedrick M.P. (2001). A simple, high-resolution method for establishing DNA binding affinity and sequence selectivity. J. Am. Chem. Soc..

[93] Miao Y., Cui T., Leng F., Wilson W.D. (2008). Inhibition of high-mobility-group A2 protein binding to DNA by netropsin: A biosensor-surface plasmon resonance assay. Anal. Biochem..

[94] Abu-Daya A., Brown P.M., Fox K.R. (1995). DNA sequence preferences of several AT-selective minor groove binding ligands. Nucleic Acids Res..

[95] Wang L., Bailly C., Kumar A., Ding D., Bajic M., Boykin D.W., Wilson W.D. (2000). Specific molecular recognition of mixed nucleic acid sequences: An aromatic dication that binds in the DNA minor groove as a dimer. Proc. Natl. Acad. Sci. USA.

[96] Wang L., Carrasco C., Kumar A., Stephens C.E., Bailly C., Boykin D.W., Wilson W.D. (2001). Evaluation of the influence of compound structure on stacked-dimer formation in the DNA minor groove. Biochemistry.

[97] Bailly C., Tardy C., Wang L., Armitage B., Hopkins K., Kumar A., Schuster G.B., Boykin D.W., Wilson W.D. (2001). Recognition of ATGA sequences by the unfused aromatic dication DB293 forming stacked dimers in the DNA minor groove. Biochemistry.

[98] Wang L., Kumar A., Boykin D.W., Bailly C., Wilson W.D. (2002). Comparative thermodynamics for monomer and dimer sequence-dependent binding of a heterocyclic dication in the DNA minor groove. J. Mol. Biol..

[99] Balthasart F., Plavec J., Gabelica V. (2013). Ammonium ion binding to DNA G-quadruplexes: Do electrospray mass spectra faithfully reflect the solution-phase species?. J. Am. Soc. Mass Spectrom..

[100] Riccardi-Sirtori F., Aldini G., Colombo M., Colombo N., Malyszko J., Vistoli G., D’Alessio R. (2012). Molecular recognition of T:G mismatched base pairs in DNA as studied by electrospray ionization mass spectrometry. ChemMedChem.

